# Targeted Achilles Tendon Training and Rehabilitation Using Personalized and Real-Time Multiscale Models of the Neuromusculoskeletal System

**DOI:** 10.3389/fbioe.2020.00878

**Published:** 2020-08-12

**Authors:** Claudio Pizzolato, Vickie B. Shim, David G. Lloyd, Daniel Devaprakash, Steven J. Obst, Richard Newsham-West, David F. Graham, Thor F. Besier, Ming Hao Zheng, Rod S. Barrett

**Affiliations:** ^1^School of Allied Health Sciences, Griffith University, Gold Coast, QLD, Australia; ^2^Griffith Centre of Biomedical and Rehabilitation Engineering, Menzies Health Institute Queensland, Griffith University, Gold Coast, QLD, Australia; ^3^Auckland Bioengineering Institute, The University of Auckland, Auckland, New Zealand; ^4^School of Health, Medical and Applied Sciences, Central Queensland University, Bundaberg, QLD, Australia; ^5^Department of Health and Human Development, Montana State University, Bozeman, MT, United States; ^6^Centre for Orthopaedic Translational Research, School of Surgery, The University of Western Australia, Nedlands, WA, Australia

**Keywords:** biomechanics, strain, mechanobiology, adaptation, Achilles tendon

## Abstract

Musculoskeletal tissues, including tendons, are sensitive to their mechanical environment, with both excessive and insufficient loading resulting in reduced tissue strength. Tendons appear to be particularly sensitive to mechanical strain magnitude, and there appears to be an optimal range of tendon strain that results in the greatest positive tendon adaptation. At present, there are no tools that allow localized tendon strain to be measured or estimated in training or a clinical environment. In this paper, we first review the current literature regarding Achilles tendon adaptation, providing an overview of the individual technologies that so far have been used in isolation to understand *in vivo* Achilles tendon mechanics, including 3D tendon imaging, motion capture, personalized neuromusculoskeletal rigid body models, and finite element models. We then describe how these technologies can be integrated in a novel framework to provide real-time feedback of localized Achilles tendon strain during dynamic motor tasks. In a proof of concept application, Achilles tendon localized strains were calculated in real-time for a single subject during walking, single leg hopping, and eccentric heel drop. Data was processed at 250 Hz and streamed on a smartphone for visualization. Achilles tendon peak localized strains ranged from ∼3 to ∼11% for walking, ∼5 to ∼15% during single leg hop, and ∼2 to ∼9% during single eccentric leg heel drop, overall showing large strain variation within the tendon. Our integrated framework connects, across size scales, knowledge from isolated tendons and whole-body biomechanics, and offers a new approach to Achilles tendon rehabilitation and training. A key feature is personalization of model components, such as tendon geometry, material properties, muscle geometry, muscle-tendon paths, moment arms, muscle activation, and movement patterns, all of which have the potential to affect tendon strain estimates. Model personalization is important because tendon strain can differ substantially between individuals performing the same exercise due to inter-individual differences in these model components.

## Introduction

The human Achilles tendon is a complex three-dimensional structure that enhances power production and efficiency of the triceps surae muscle-tendon complex during movement (Lichtwark and Wilson, 2005; Handsfield et al., 2017; Pekala et al., 2017; Wiesinger et al., 2017). The Achilles tendon experiences strain when the triceps surae muscle force increases, and the energy stored in the tendon can be rapidly recovered during subsequent unloading, thereby facilitating efficient locomotion. In extreme cases, the Achilles tendon can completely rupture, but more commonly undergoes tendinopathic changes and symptoms, which include reduced Young’s modulus, focal pain, and associated motor dysfunction (Paavola et al., 2002; Arya and Kulig, 2010; Obst et al., 2018; McAuliffe et al., 2019). It is currently difficult to predict individuals at risk of tendon injury, and the individual responses to an exercise-based Achilles tendon rehabilitation protocol. Changes in mechanical properties and geometry of tendon have been reported as a response to mechanical stimuli (Arampatzis et al., 2010). However, the mechanical stimuli provided to the tendon during exercise or rehabilitation cannot be estimated from external biomechanics (Pizzolato et al., 2017a), which possibly explains why the loading dose and modality of training protocols for the management of tendinopathy remain unclear (Wilson et al., 2018).

In this article, we first provide a brief overview of Achilles tendon biomechanics, mechanobiology, and adaptation. We then propose an integrated framework to better understand localized mechanical environment of the tendon during motor tasks, which is required to help prevent Achilles tendon damage/injury and enhance outcomes from Achilles tendon training and rehabilitation. Finally, we provide a proof of concept application of our framework by estimating localized strain within the Achilles tendon in real-time during walking, single leg hopping, and eccentric heel drop exercises.

## Achilles Tendon Biomechanics, Mechanobiology, and Adaptation

Musculoskeletal tissues are sensitive to their mechanical environment, with both excessive and insufficient loading resulting in reduced tissue strength (Wang et al., 2013, 2015). Whereas bone appears to be most sensitive to loading at high strain rates (Turner et al., 1995; Hsieh and Turner, 2001; Hart et al., 2017), tendon instead appears to be particularly sensitive to the magnitude of tissue strain (Wang, 2006; Heinemeier and Kjaer, 2011; Galloway et al., 2013). *In vitro* bioreactor studies in rabbit Achilles tendons revealed tendons that experienced strain magnitudes of 6% at 0.25 Hz for 8 h per day for 6 days had greater expression of type I collagen (characterized by high stiffness and superior mechanical properties) and lower expression of type III collagen (characterized by inferior mechanical properties) than tendons that experienced either 3% or 9% strains at the same duration and frequency (Wang et al., 2013). Furthermore, tendons that were load-deprived for 12 days experienced increased type III collagen expression and had reduced mechanical strength, but were able to increase type I collagen expression and material properties following a loading regime that produced 6% strain (6 days, 8 h/day, 0.25 Hz) (Wang et al., 2015). *In vivo* studies have also systematically manipulated Achilles tendon loading parameters including strain magnitude, duration, rate, and frequency. For example, Arampatzis et al. (2010) reported an increase in elastic modulus of the Achilles tendon following isometric plantar flexor training (20 repetitions, 5 sets/session, 4 days/week) performed at a strain magnitude of ∼5%, and no improvement when the same training total volume was performed at a lower strain magnitude (∼3%). The same authors also reported that an increase in strain rate from 0.17 to 0.5 Hz resulted in only a moderate improvement of Achilles tendon mechanical properties (Arampatzis et al., 2010). A systematic review of human tendon adaptation in response to mechanical loading further concluded that, on the basis of 27 included studies, while tendons are responsive to a range of loading conditions, loading magnitude (and hence strain), in particular, plays a key role in tendon adaptation (Bohm et al., 2015). While there remains much to learn about the specific loading conditions (i.e., strain magnitude, strain duration, strain frequency, and strain rate) that maximize tendon adaptation and the mechanobiological pathway involved (Smith et al., 2013; Young et al., 2016), tendon strain magnitude appears to play a fundamental role.

Based on the presumption that strain magnitude is a key control variable for tendon adaptation, it follows that the efficacy of exercise-based training and rehabilitation programs might be improved by ensuring that the strain magnitude experienced by the Achilles tendon during exercise is in the anabolic range of approximately 5–6%, where tendon remodeling exceeds strain-induced tendon damage (Pizzolato et al., 2019). However, there are no tools available that allow localized tendon strain to be measured or estimated in either the training or clinical environment. Arguably, the best non-invasive method of direct measurement available at present involves attaching an ultrasound probe to the leg to track the motion of the gastrocnemius muscle-tendon junction and estimating the change in tendon length relative to a motion capture marker placed on the calcaneus (Stenroth et al., 2012; Zelik and Franz, 2017). While this approach has provided new and important insight into the strains experienced during tasks such as walking, running and jumping, the approach remains technically challenging and impractical outside of the laboratory environment. Alternative approaches to strain mapping of the Achilles tendon during functional movements are therefore required.

In this article, we propose and demonstrate a framework that combines 3D tendon imaging, motion capture, and personalized neuromusculoskeletal (NMS) rigid body and finite element (FE) models to assess the localized stress and/or strain in the free Achilles tendon (region spanning from the calcaneal insertion to soleus muscle tendon junction) for the purpose of providing instantaneous feedback during dynamic motor tasks. A key feature of the framework is personalization of model features with the potential to affect calculation of strain, which include tendon geometry and material properties, muscle geometry and muscle-tendon paths and/or moment arms, and muscle activation and movement patterns. This is important because tendon stress and/or strain could differ substantially between individuals performing the same exercise prescription such as eccentric heel drops used in treatment of tendinopathy (Alfredson et al., 1998), even if they are matched for age, size, gender, training history, and clinical status. The proposed framework will help to clarify the optimum loading dose for training and rehabilitation of the Achilles tendon by facilitating exercise prescription based on internal tissue loading.

## Framework for Real-Time Estimation of Localized Achilles Tendon Stress and Strain

**FIGURE 1:**
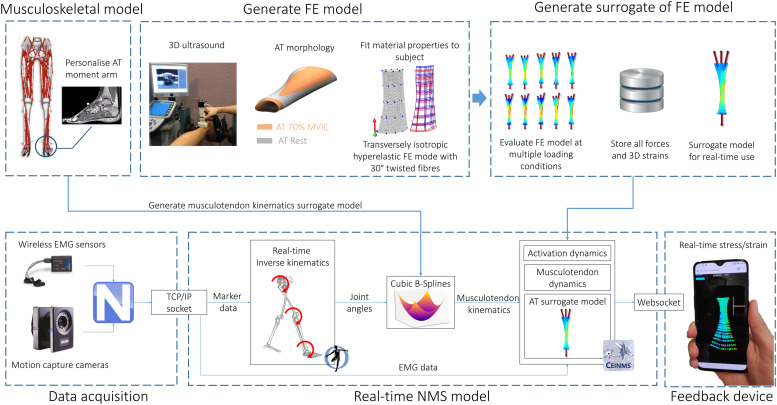
Model creation and data processing pipeline. A generic OpenSim musculoskeletal model (gait2392) was linearly scaled to the individual using motion capture data. The moment arm of medial gastrocnemius, lateral gastrocnemius, and soleus muscle-tendon units were adjusted to match the Achilles tendon moment arm measured via magnetic resonance imaging. FE model of the Achilles tendon was created from 3D ultrasound following established methods. A surrogate of the FE model was used to predict localized strains at 2048 Gauss points throughout the Achilles tendon. Joint angles were calculated via real-time inverse kinematics. Muscle-tendon lengths, calculated via multidimensional cubic B-splines, and surface EMG were used to drive a Calibrated EMG-informed NMS model (CEINMS) in open-loop. Muscle forces predicted by CEINMS were used as input for the Achilles tendon surrogate model, enabling estimation of localized strains. Real-time visual feedback of strain was implemented in Javascript three.js (r100) and visualized on a mobile phone via internet browser (Mozilla Firefox).

The proposed framework for estimating Achilles tendon stress and strain in real-time involves integration of motion capture, electromyography, medical imaging, and NMS and FE models (Figure 1). Body kinematics and muscle activation patterns are used as inputs into a rigid body electromyogram (EMG) – informed NMS model that computes the triceps sure muscle forces applied to the Achilles tendon. These muscle forces are subsequently applied to a personalized FE model of the Achilles tendon based on geometry and material properties determined from a combination of 3D medical imaging, isometric measurements, and model fitting. Real-time estimates of Achilles tendon stress and/or strain are enabled by the use of a surrogate FE model that is first generated offline, and then solved for a feasible set of muscle forces. The corresponding stress/strain calculated via the personalized Achilles tendon FE model are then used to create a surrogate model via polynomial fitting, regression methods, or other machine learning approaches. Each of these steps is described in detail in the following sections.

### Personalized Achilles Tendon 3D Geometry and Deformation From Freehand 3D Ultrasound

Conventional 2D brightness-mode (b-mode) ultrasound has been widely used to quantify the length, thickness, and cross-sectional area of the Achilles tendon. 2D b-mode ultrasound may also be used to create 3D reconstruction of the Achilles tendon when used in conjunction with motion capture (Devaprakash et al., 2019). This approach, termed freehand 3D ultrasound, involves simultaneously recording the position and orientation of the ultrasound probe in 3D space as it is swept along the free Achilles tendon. The generated stack of 2D images is then positioned and orientated in a global coordinate system. Image slices are then segmented to generate a volumetric reconstruction of the Achilles tendon. Estimates of free Achilles volume, length, and cross-sectional area from freehand 3D ultrasound have been shown to be both highly repeatable (Obst et al., 2014a) and in close agreement with corresponding estimates obtained from high resolution magnetic resonance imaging (MRI) scans (Devaprakash et al., 2019). The main limitation of freehand 3D ultrasound is that the free Achilles tendon must remain stationary during the freehand scan and so the method is confined to resting or isometric conditions. However, unlike MRI, freehand 3D ultrasound allows macroscopic Achilles tendon geometry to be readily measured in the laboratory environment at rest and during voluntary isometric loading.

Experimental studies to date using freehand 3D ultrasound have demonstrated that longitudinal strain of the free Achilles tendon is more than double the longitudinal strain experienced by the proximal aponeurosis (Obst et al., 2016), and that the free Achilles tendon experiences twist during loading (Obst et al., 2014b). Studies of 3D tendon deformation during and following tendon conditioning (Nuri et al., 2016, 2018a) and following eccentric heel drop exercise (Obst et al., 2015, 2016) have further confirmed that changes in strain behavior under a constant isometric load are primarily confined to the free Achilles tendon, rather than the proximal tendon or the aponeurosis. In contrast to the healthy free Achilles tendon, which becomes narrower along the medial-lateral axis and bulges along the anterior-posterior axis during longitudinal deformation, the tendinopathic Achilles tendon experiences deformation along both transverse plane axes, as well as a corresponding volume reduction during sustained longitudinal deformation (Nuri et al., 2017, 2018b). Geometry and 3D deformation of the Achilles tendon under loading condition can be used create personalized FE models and estimate localized stress/strain.

### Personalized Finite Element Models of the Achilles Tendon

**FIGURE 2:**
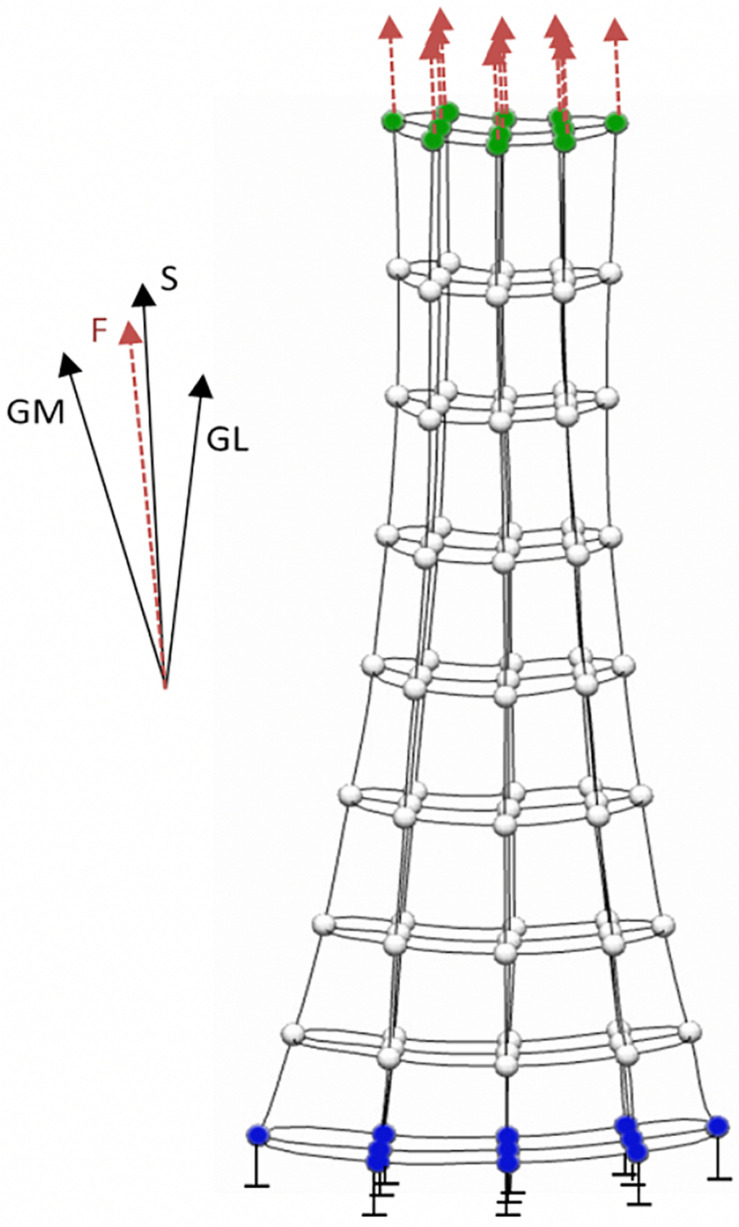
Boundary conditions applied to the finite element model of the Achilles tendon. The model’s distal nodes (blue) are constrained and not allowed to move. Boundary force from gastrocnemius medialis (GM), gastrocnemius lateralis (GL) and soleus (S) is equally distributed on the proximal nodes (green) of the model.

Finite element analysis allows the localized tissue stress or strain to be computed as a function of the personalized tissue geometry and material properties in response to externally applied loads (i.e., boundary conditions). Prior to FE analysis, a generic tendon mesh is morphed to match the resting 3D geometry of tendon measured using freehand 3D ultrasound via free form deformation (Fernandez et al., 2004). The constitutive mechanical behavior of the free Achilles tendon can be modeled using a transversely isotropic hyperelastic formulation, which has been successfully applied to both cadaveric (Shim et al., 2014) and *in vivo* analyses (Hansen et al., 2017; Shim et al., 2018, 2019). Material properties of the Achilles tendon can be estimated using an inverse FE approach, wherein an optimization procedure identifies the subject-specific material properties parameters that produce the best match between the geometry of the loaded subject-specific FE mesh and the geometry of the free Achilles tendon measured under submaximal loading conditions using freehand 3D ultrasound (Hansen et al., 2017). Achilles tendon forces required as boundary conditions during the inverse FE approach (Figure 2) can be readily calculated from measured angle joint moment and Achilles tendon moment arm. After this procedure, the personalized FE model of the Achilles tendon can estimate all principal component stress/strains, which can be re-aligned with anatomical directions (i.e., proximal-distal, medial-lateral, and anterior-posterior) (Shim et al., 2018), for a range of triceps sure forces.

In the healthy free Achilles tendon, stress is more sensitive to subject-specific differences in tendon geometry than subject-specific differences in tendon material properties (Hansen et al., 2017). The location of peak tendon stress and the predicted rupture location vary considerably between cadaveric tendon samples under equivalent loading conditions (Shim et al., 2014). As both tendon geometry and material properties (Arya and Kulig, 2010; Obst et al., 2018) are altered in Achilles tendinopathy, it is also important to determine how these alterations affect Achilles tendon stress distributions in tendinopathic Achilles tendons. Shim et al. (2019) reported that tendinopathic tendons have a 30% larger cross-sectional area and 50% lower modulus compared to healthy tendons, but that the lower modulus resulted in only an 8% increase in average tendon stress during a 70% submaximal isometric contraction, compared to a 30% decrease in average tendon stress due to the larger cross-sectional area. It was therefore suggested that the larger cross-sectional area reported in tendinopathic Achilles tendons (Arya and Kulig, 2010) could be protective against higher tendon stresses, but could also result in regions of underloading/stress shielding with associated catabolic effects on the tendon.

The aforementioned FE models of the Achilles tendon were developed from the macroscopic 3D tendon geometry assessed using freehand 3D ultrasound, and therefore do not incorporate more detailed substructural Achilles tendon geometry such as the twisted substructure of individual sub-tendons (Edama et al., 2015; Pekala et al., 2017) and the proximal aponeurosis. By including fascicle twist in a FE model of the human Achilles tendon, Shim et al. (2018) demonstrated that twist increased the predicted rupture load by 40%, and that ∼30° minimized tendon stress, with higher and lower twist angles resulting in higher tendon stress. Future Achilles tendon models should include more detailed substructural estimates of spatial distribution of material properties. While there is some initial evidence that a tendinopathic inclusion alters the stress distribution in the vicinity of the lesion (Maganaris et al., 2017), further research is required to more fully characterize the mechanical environment in and around the lesion in tendinopathy.

A current limitation of FE analysis in the context of the proposed use as a biofeedback tool, is that FE models are computationally intensive and therefore not amenable to real-time application. This is circumvented by first solving the FE model offline for a large number of feasible triceps sure muscle force combinations, then use machine learning approaches (sometimes called surrogate methods), such as partial least squares regression or polynomial fitting, to estimate the tendon stress/strain distribution in real-time with minimal computational requirements.

### Personalized Real-Time Neuromusculoskeletal Models

Neuromusculoskeletal models are causal physics-based representations of an individual’s bones, muscles, tendons, and joints. The skeletal structure is represented as a rigid multi-body system. Muscles are modeled as Hill-type actuators, and joints are subjected to mechanical constraints that represent their real counterpart (Delp et al., 2007). Motion capture (e.g., stereophotogrammetry marker-based systems or inertial measurement units) and external force data (e.g., ground reaction forces) are used to determine joint angles and moments using inverse kinematics and inverse dynamics approaches, respectively. Mechanical methods based on metabolic expenditure optimization criterion, such as static or dynamic optimization, can be used to solve the muscle redundancy problem; however, these approaches do not account for the individual’s motor control and have repeatedly shown to be unable to appropriately predict muscle co-contractions that are evident in measured EMG data (Hoang et al., 2018; Davico et al., 2019b; Veerkamp et al., 2019). Alternatively, EMG data can be directly used to inform forward dynamic simulations of muscles contraction and calculate muscle forces (Lloyd and Besier, 2003; Pizzolato et al., 2015, 2017c). When personalized to the individual (e.g., via system identification methods or direct measurement) EMG-informed NMS models can predict physiologically plausible muscle forces across different motor tasks and different populations varying by age and pathology (Gerus et al., 2013; Hoang et al., 2018; Davico et al., 2019b; Veerkamp et al., 2019).

Personalization of NMS models may involve musculoskeletal geometry (e.g., bone shape and dimensions, muscle-tendon insertion points and pathways, joint mechanics) and neuromuscular parameters (e.g., maximum isometric forces of muscles, tendon slack length, optimal fiber length, activation dynamics) (Saxby et al., 2020). A rudimentary form of musculoskeletal geometry personalization is linear scaling of bone geometry based on discrete anthropometric measurements. While this approach is easy to implement, the resulting geometry may poorly represent the individual, affecting muscle-tendon attachment points and pathways, and consequent estimation of muscle-tendon forces (Gerus et al., 2013; Bahl et al., 2019). More advanced and promising methods use medical imaging databases to build population-based statistical shape models (Zhang et al., 2014; Zhang and Besier, 2017; Davico et al., 2019a). Sparse data, such as anatomical landmarks, of individuals not previously included in the database are then used to reconstruct the geometry of bones. In the context of the Achilles tendon, statistical shape models of primary foot bone segments, as well as tibia-fibula and femur, can be reconstructed with minimal error (Davico et al., 2019a; Grant et al., 2020), but future work should investigate whether these accurate predictions of bone geometry are also reflected in accurate predictions of Achilles tendon moment arm.

Neuromuscular parameters affect the amplitude and timing of predicted muscle-tendon forces and personalization has been shown essential in predicting physiologically plausible muscle and joint contact forces (Hoang et al., 2018). While some parameters, such as maximum isometric force of individual muscle, can be inferred from medical imaging data (O’Brien et al., 2010; Handsfield et al., 2014), optimization algorithms are required to calibrate parameters that cannot be observed (Pizzolato et al., 2015; Hoang et al., 2018). Calibration involves minimizing the error between the joint moments estimated from EMG-informed NMS models and from inverse dynamics, while satisfying a variety of physiological constraints (e.g., muscle fascicles working within a plausible range of their force-length characteristic) (Pizzolato et al., 2015). Including a variety of different tasks and trials in the calibration also ensures that the NMS model is appropriately calibrated within a large solution space (Lloyd and Besier, 2003; Saxby et al., 2016). After calibration, the NMS model can predict novel trials and tasks for the same subject.

Recent advances in NMS modeling have also made it possible to execute EMG-informed NMS models in real-time (Manal et al., 2012; Pizzolato et al., 2017c; Durandau et al., 2018), enabling instantaneous estimates of the state of internal musculoskeletal tissues. This has extended the potential application of physiologically sound models to training and rehabilitation (Pizzolato et al., 2017a); a feat previously considered unattainable. This technology has been used to estimate internal knee loading during gait, showing that a person can change their gait patterns to volitionally modulate the amplitude of their knee contact force (Pizzolato et al., 2017c), and to estimate the amount of force experienced by the Achilles tendon during post-rupture rehabilitation (Manal et al., 2012). These prior examples of real-time estimation of internal biomechanics have been limited to forces alone, which only partially represent the mechanical stimuli responsible for tissue adaptation (Pizzolato et al., 2017a). Recent advances in computational rigid body NMS and FE modeling has resulted in technologies that are sufficiently mature to be combined into personalized real-time multi-scale models of the person and their tissues. We present a proof of concept of this integrated technology applied to estimation of free Achilles tendon localized strain during dynamic motor tasks. We show that the developed multiscale model can calculate the strain field within the free Achilles tendon in real-time and with minimal computational effort using as input EMG and motion capture data alone. Future refinement of this technology should involve rapid creation of personalized rigid-body and FE models to create a clinic-ready system for advanced training and rehabilitation.

## Proof of Concept Application

One healthy individual (age: 21, mass: 70.5 kg, height: 1.75 m) gave written informed consent to participate in this study (Griffith University ethics approval number 2017/020).

### Experimental Setup and Data Acquisition

Prior to all testing, the participant followed a standardized protocol for Achilles tendon preconditioning (Hawkins et al., 2009). Anatomical MRI scans of the ankle joint, including foot and distal tibia-fibula, were acquired on a Philips Ingenia 3.0 Tesla scanner (Amsterdam, Netherlands) using an 8-channel ankle coil (PDW 3D TSE, TR/TE 1000/41 ms) with the participant lying in a supine position with the hip in neutral position and knee fully extended. The foot was supported inside the coil by a pad to ensure that the ankle was in a neutral position (0° dorsiflexion).

Achilles tendon geometry measurements were performed as follows. The participant was positioned prone on a bed with their foot firmly secured to the dynamometer foot plate, which was locked with the ankle in a neutral position (0°). The knee joint was fully extended, and the hip joint was in neutral position. Freehand 3D ultrasound data were acquired using a 2D ultrasound (Aplio 500, Canon Medical Systems, Otawara, Japan) and motion capture system (Vicon MX T-series, Vicon Motion Systems Ltd., Oxford, United Kingdom), as described in Devaprakash et al. (2019). A dynamometer (HUMAC NORM, Stoughton, MA, United States) was used to concurrently measure ankle plantarflexion torque. All equipment was synchronized via a hardware trigger. After preconditioning, freehand 3D ultrasound data of the Achilles tendon was collected at rest and at 25, 50, and 70% of maximum voluntary isometric contraction (MVIC). Measurements were repeated three times per condition.

A 20-camera motion capture system (Vicon, Oxford, United Kingdom) and 8 force plates (Kistler Instrument Corporation, Amherst, NY, United States) were used to collect marker trajectories (250 Hz) and ground reaction forces (1000 Hz) during walking, running, single leg hopping, and eccentric heel drops. Surface EMG (TELEmyo DTS, Noraxon U.S.A. Inc., Scottsdale, AZ, United States) were also acquired (1500 Hz) from 16 sites on a single leg: medial gastrocnemius, lateral gastrocnemius, soleus, flexor digitorum hallucis longus, peroneus longus, peroneus brevis, tibialis anterior, extensor hallucis longus, vastus medialis, vastus lateralis, rectus femoris, semitendinosus, biceps femoris, sartorius, tensor fascia latae, and gracilis. All the systems were synchronized via hardware trigger.

### Neuromusculoskeletal and Finite Element Models

A generic OpenSim (Delp et al., 2007) model (gait2392) was linearly scaled to the individual. The moment arms of the medial and lateral gastrocnemius and soleus muscles were automatically adjusted in the scaled OpenSim model in order to reflect the experimental measure from MRI data (Alexander et al., 2017). This was achieved by optimizing the distal attachment point of the three musculotendon units. Multidimensional cubic B-splines were used to create a surrogate model of the model’s musculoskeletal geometry for real-time estimation of muscle-tendon lengths and moment arms (Sartori et al., 2012).

Experimental free Achilles tendon elongations were calculated from isometric freehand 3D ultrasound measurements, as the distance between the calcaneal notch and soleus muscle-tendon junction. Isometric ankle torque data and Achilles tendon moment arm experimentally measured from MRI were then combined with tendon elongation measurements to calculate normalized force-elongation points. The following piecewise function (Schutte, 1993)

$$\left\{ \begin{array}{cc} f\,\left(\varepsilon \right)=0, & \varepsilon <0\\ f\,\left(\varepsilon \right)=a\,\varepsilon^{2}, & \varepsilon <\varepsilon_{0}\\ f\,\left(\varepsilon \right)=m\,\varepsilon +q, & \varepsilon \geq \varepsilon_{0} \end{array}\right.$$

was fitted to the experimental values by optimizing a, m, q, and ε_*0*_ while ensuring C^1^ continuity, where *f* represents force and ε strain. This resulted in a continuous dimensionless curve that was applied to tendon of the medial gastrocnemius, lateral gastrocnemius, and soleus muscle-tendon units in the NMS model. Tendon force elongation curves for the other muscle-tendon units in the model were based on literature data (Millard et al., 2013). Maximum isometric forces for each of the triceps surae muscles were estimated from MRI measured muscle volumes and muscle optimal fiber lengths (from gait2392 OpenSim model) using a specific tension of 55 N/cm^3^ (O’Brien et al., 2010). The remaining NMS model parameters were then calibrated via established methods (Pizzolato et al., 2015) using one trial from each experimental dynamic task in the calibration procedure. Optimized parameters included optimal fiber length, tendon slack length, and maximum isometric forces of the muscles not measured, as well as muscle activation/deactivation time constants (Pizzolato et al., 2015).

A FE model of the free Achilles tendon was modeled using CMISS^1^ as an incompressible, transversely isotropic hyperelastic material with 30° twisted fibers, and geometry personalized via freehand 3D ultrasound, as well as material properties parameters calculated via inverse FE approaches (Shim et al., 2014, 2019; Hansen et al., 2017). To enable calculation of longitudinal principal component strains (i.e., localized), the reference FE material coordinate system was rotated to align with the fiber orientation of the tendon, which run in the proximal-distal direction (Shim et al., 2018). The material behavior of the model was defined via six coefficients (Hansen et al., 2017), C1 to C6. Coefficients C2 was set from literature (C2 = 0 to describe a Neo-Hookean material). Coefficient C5, corresponding to Young’s modulus, was calculated from experimental 3D ultrasound data as the ratio between tendon stiffness and average free Achilles tendon cross-sectional area (C5 = 0.58 GPa). Coefficients C1, C3 (scaling of the exponential stress), and C4 (rate of collagen fiber loading) were optimized by minimizing the root mean square error (RMSE) between the longitudinal tendon elongation in the FE model under load and the corresponding measured experimental tendon elongation at 70% MVIC. Finally, coefficient C6 was calculated as function of C2, C4, and C5 (Hansen et al., 2017). After creating the personalized FE model of the free Achilles tendon, localized strains were calculated for 2048 Gauss points (i.e., integration points) by solving the FE problem for a range of boundary conditions. Specifically, the model was constrained at the distal end and forces that ranged from 0 to 200% MVIC at 10% intervals were evenly applied to the surface of the proximal end of the tendon, in the direction corresponding to the longitudinal (i.e., proximal-distal) axis of inertia of the tendon. This resulted in a total of 21 localized strain data points per Gauss point that were a function of the force applied as boundary condition, which were then used as training data for the surrogate FE model. Three different surrogate methods were assessed in their ability to reproduce FE data (RMSE; coefficient of correlation, R) using a leave-one-out validation. The three tested models were: partial least square regression, cubic spline interpolation, and basis spline interpolation. For both the spline-based models, one spline per Gauss node (*n* = 2048) was used to interpolate the localized strain as a function of the force applied to the free Achilles tendon.

### Real-Time Estimation of Achilles Tendon Strain

Real-time conditions were simulated using Vicon Virtual System (v1.4.1), which allows emulating the connection with live streaming cameras and analog devices. Vicon Nexus 2.8 was used to acquire the stream and perform automatic and real-time marker labeling. Labeled, but unfiltered, marker and EMG data were streamed to a custom, multithreaded software pipeline implemented in C++. The software pipeline (Figure 1) used real-time components previously described and validated (Pizzolato et al., 2017b, c) that allowed for computationally efficient estimation of real-time muscle-tendon forces, and it was extended to include surrogate FE model of the free Achilles tendon. In brief, marker data was used to estimate joint angles using a real-time OpenSim inverse kinematics algorithm (Pizzolato et al., 2017b), which were then filtered and used to estimate muscle-tendon kinematics (i.e., lengths and moment arms) of the lower limb. Musculotendon kinematics and surface EMG were then used to drive a three-degrees of freedom (knee flexion extension, ankle plantar-dorsiflexion and pronation-supination), calibrated EMG-informed NMS model in open-loop using CEINMS software (Pizzolato et al., 2015). Predicted triceps surae muscle forces were summed and used in input for the surrogate model of the free Achilles tendon, enabling estimation of localized strains. A real-time visual feedback of strain was implemented in Javascript three.js (r100) and visualized in a web browser (Mozilla Firefox) on a mobile phone (OnePlus 6T, OnePlus) via websocket (Figure 1). Computations were all performed on a laptop workstation (Windows 7, i7-6820HQ @ 2.7 Hz, 32 GB RAM).

Free Achilles tendon localized strains during walking, single leg hopping, and eccentric heel drop, as well as real-time performance were reported. The real-time pipeline was also assessed to correctly function in real laboratory conditions, but due to the unavailability of the same subject used to generate the personalized models, only simulated real-time data were reported.

### Results

**FIGURE 3:**
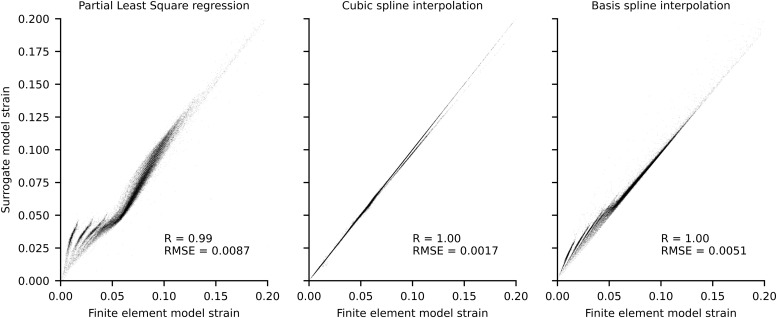
Scatterplots of localized strains of each Gauss point for finite element model and surrogate models. Data was produced using a leave one out procedure, wherein each surrogate model was trained n times on n-1 conditions and used to predict the condition not included in the training. The force boundary condition used to execute the finite element model ranged between 0 and 2 times the experimental maximum voluntary isometric contraction (MVIC), at 0.1 MVIC increments, which resulted in *n* = 21.

The surrogate model based on cubic splines outperformed both partial least square regression and basis spline interpolation in the leave-one-out validation (Figure 3) and was consequently used in the real-time pipeline to calculate Achilles tendon strain.

**FIGURE 4:**
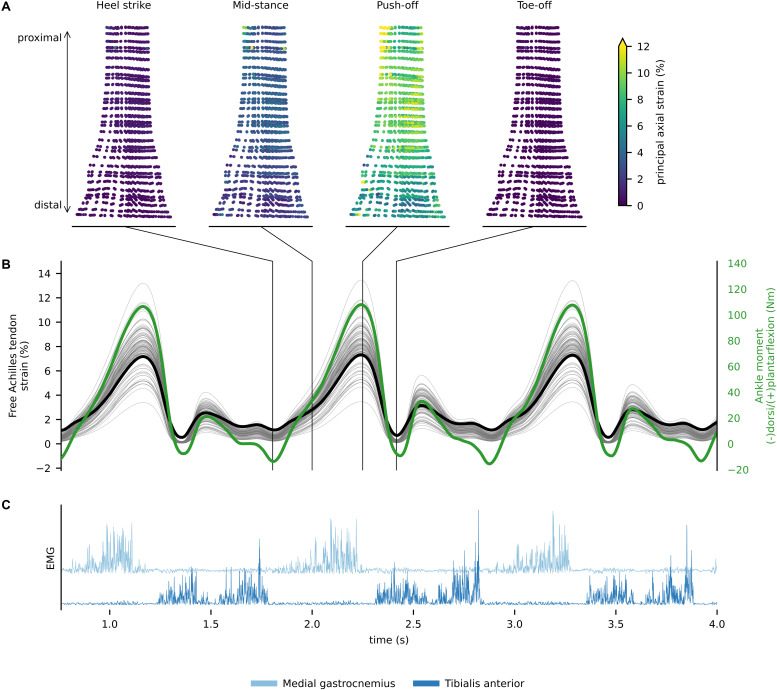
**(A)** *In vivo* free Achilles tendon localized strains for discrete events of the gait cycle. **(B)** Free Achilles tendon global (thick black line), localized strain (thin gray lines) for each Gauss point, and ankle moment (green line) during walking. **(C)** Rectified electromyograms of tibialis anterior and medial gastrocnemius showing phasic muscle activity during walking.

**FIGURE 5:**
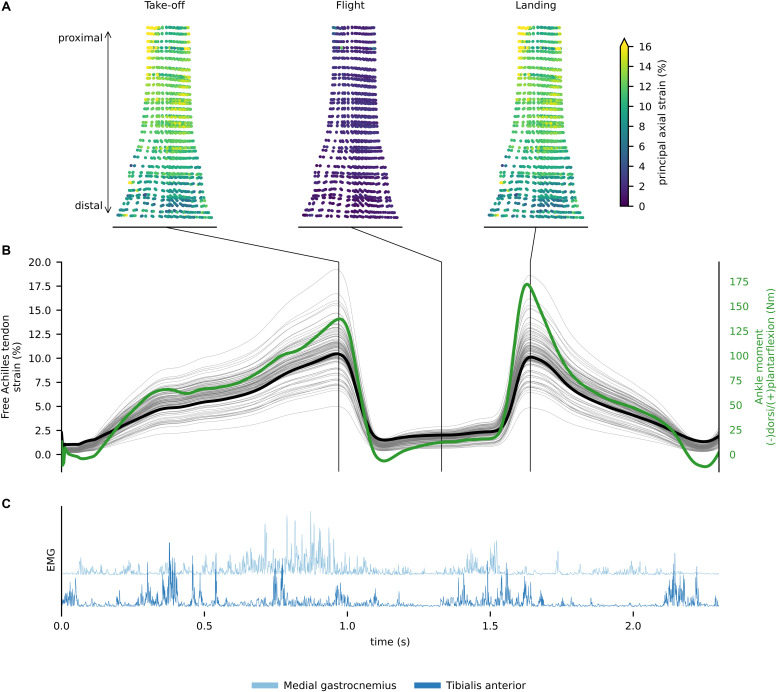
**(A)** *In vivo* free Achilles tendon localized strains for discrete events of the single leg hoping. **(B)** Free Achilles tendon global (thick black line), localized strain (thin gray lines) for each Gauss point, and ankle moment (green line) during single leg hopping. **(C)** Rectified electromyograms of tibialis anterior and medial gastrocnemius.

**FIGURE 6:**
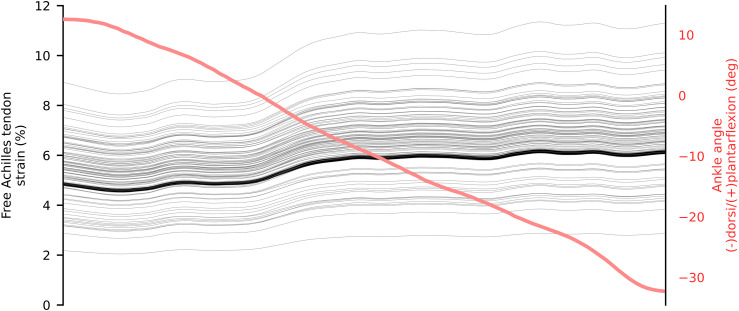
Mean *in vivo* free Achilles tendon global (thick black) and localized (thin gray) strain from 5 repetition of eccentric heel drop. The ankle angle is represented in red.

In walking, the greatest amount of free Achilles tendon strain was present in the push-off phase, which also corresponded to the production of maximal plantar-flexion moment. At push-off, the localized strains within the tendon varied from ∼3 to ∼11%, while global strain (proximal to distal elongation normalized by resting length) was ∼6.5% (Figure 4). During single leg hopping, maximum strain also corresponded to the production of maximal plantar-flexion moment, but greater plantarflexion torque in the landing phase compared to the take-off phase did not correspond to greater free Achilles tendon strains. During these phases, localized strains within the tendon varied from ∼5 to ∼15%, while global strain was ∼10% (Figure 5). The greatest magnitude of localized strain was present in the mid-portion and on the proximal medial side of the free Achilles tendon. During eccentric heel drops, localized strains within the tendon varied on average from ∼2 to ∼9%, increasing of ∼1% in full dorsiflexion, while the global free Achilles tendon strain peaked at ∼6% (Figure 6).

**FIGURE 7:**
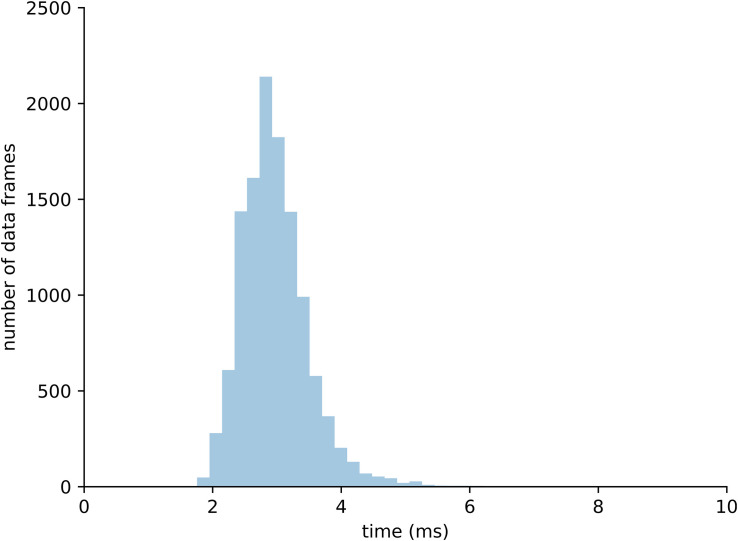
Distribution of computational time required to process 10,000 data frames within the complete real-time pipeline.

A total of 10,000 data frames computed in real-time were analyzed to assess performance of the integrated system. Each data frame was computed on average in 3.02 ms, which is 1.3 times faster than the time sampling of experimental marker data from the motion capture system (i.e., 250 Hz or 4 ms). Of all analyzed data frames, 95% were computed in less than 3.86 ms, which includes all the calculations from raw marker data to tendon strain computation (Figure 7).

## Discussion and Future Directions

This paper demonstrates proof of concept that an integrated technology-based approach may be used to provide real-time feedback in a training and/or rehabilitation setting to ensure that a targeted dose of mechanical stimuli is delivered to the musculoskeletal tissue. We have demonstrated that it is possible to estimate localized tissue strain in the free Achilles tendon during walking, hopping and eccentric heel drops, in real-time using an EMG-informed NMS model combined with a surrogate FE model. While some further development and validation of our framework is required, we foresee that in the near future it will be possible to provide instantaneous feedback of Achilles tendon tissue strain in a clinical or training environment, thereby facilitating personalized training and/or rehabilitation of the Achilles tendon that targets the loading range that elicits positive tendon adaptation (hypertrophy).

Using standard laboratory-based equipment, we have demonstrated it is possible to estimate Achilles tendon strain in real-time with minimal delay (less than 3.86 ms for 95% of the data), which is essential to provide appropriate and timely feedback to the user (Kannape and Blanke, 2013). Real-time biofeedback of triceps surae muscle-tendon forces (Manal et al., 2012) and tibiofemoral joint contact force (Pizzolato et al., 2017c) from EMG-informed musculoskeletal models have been previously being used to modify an individual’s gait patterns. The proposed framework takes these studies one step further by reporting localized tissue level Achilles tendon strain. As a proof of concept, we have developed a simple visual feedback interfaces that runs on a smartphone to instantly show localized Achilles tendon strain to the user (Figure 1), but we are yet to perform human-in-the-loop experiments where the person changes their training or rehabilitation to optimize tissue strains. In a simple implementation, this form of feedback could be provided to indicate when the predicted peak or average tendon strain is either within or outside the desired target range.

It is important to highlight some limitations of the proposed real-time framework. While inverse kinematics was performed in real-time using OpenSim using a previously validated method (Pizzolato et al., 2017b), marker-based motion capture may suffer from marker occlusions. As such, particular care must be taken by the experimenter to appropriately place infrared cameras and calibrate the capture volume to enable for auto labeling of markers to function correctly. Our 20-cameras setup prevented marker occlusions during the evaluated tasks, but motion capture solutions based on inertial measurement units may simplify the data acquisition setup in the future. We used multidimensional cubic B-splines to enable calculation of muscle-tendon lengths and moment arms (Sartori et al., 2012), which may appear a simplification. However, multidimensional cubic B-splines have been previously shown to be superior to polynomial fitting and to introduce negligible estimates errors for muscle-tendon kinematics (Sartori et al., 2012). Another limitation of real-time executions is the inability to use non-causal filters, such as zero-lag Butterworth filters commonly used in analysis of biomechanical data. Following previously established methods (Pizzolato et al., 2017c), we filtered EMG and joint angle data using the same causal Butterworth filter, thus minimizing errors caused by phase delays which has been shown to produce excellent agreement between real-time and offline simulations (Pizzolato et al., 2017c). Model calibration is fundamental to obtain physiologically plausible muscle-tendon forces (Hoang et al., 2018) but at the current stage this process is still computationally intensive and must be performed offline. Online calibration procedures, such as Bueno and Montano (2017), should be implemented in the future to minimize idle times. Finally, as the FE method is computationally intensive, a surrogate FE model was used to enable real-time estimation of Achilles tendon strain. Of the three evaluated methods, cubic interpolation showed the best performance, with associated strain RMSE of 0.0017. Different surrogate methods should be explored to adapt the proposed framework to more complex FE models (e.g., with subtendons). Nonetheless, prior to translation it will be necessary to conduct a comprehensive methodological evaluation of the proposed framework, including studies to determine the validity and reliability of tendon strains estimates, and sensitivity of such estimates to model parameters.

The peak global Achilles tendon strains estimated via our multiscale model (6.5% for walking and 10% for single leg hop) were higher than the maximum strain measured with 2D ultrasound from previous research (4.8% for walking and 8.2% for single leg hop) (Lichtwark and Wilson, 2005, 2006). However, previous research measured global strains of the free Achilles tendon and aponeurosis combined (i.e., calcaneal notch to medial gastrocnemius muscle-tendon junction) while we provided a strain estimate of the free Achilles tendon alone (i.e., calcaneal notch to soleus muscle-tendon junction). Importantly, longitudinal free Achilles tendon strain has been shown to be greater than the longitudinal strain of the Achilles tendon aponeurosis (Magnusson et al., 2003), which at least partially explains differences between our predicted global strains and previous measurements during dynamics tasks. Single leg hop exercises resulted in the highest free Achilles tendon global strains, which is likely related to some combination of higher muscle activation and more favorable force-length-velocity behavior (i.e., longer triceps surae muscle lengths and faster lengthening speeds) than for walking and eccentric heel drops. It is however, important to consider that we presented data from a single subject and the results are likely to differ when analyzing Achilles tendon strain distribution in multiple individuals and pathological tendons (Hansen et al., 2017; Shim et al., 2019).

The FE model used in this study has been previously employed in cadaveric experiments, demonstrating excellent ability to predict tendon rupture in quasi-static conditions (Shim et al., 2014). However, the employed FE model did not fully account for each of the individual subtendons (Pekala et al., 2017), thus preventing the prediction of differential strains between deep and superficial Achilles tendon that have been described in literature (Franz et al., 2015). In future it will be of benefit to incorporate more detailed representation of the complex substructural geometry of the tendon and localized material properties (e.g., via elastography) for normal and pathological tendons (DeWall et al., 2014). Localized AT strains ranged from 3 to 11% during walking, 5 to 15% during single leg hopping, and 2 to 9% during the eccentric heel drop and suggest that tendon strain distribution within the Achilles tendon may be highly heterogenous. The highest localized strains tended to correspond with the tendon mid-region where the tendon cross-sectional area was minimal. However, our model used a homogeneous spatial distribution of material properties and a transversely isotropic hyperelastic formulation, which does not account for the viscous behavior of human tendons. While elastic properties have been shown to prevail over viscous properties in the human Achilles tendon (Peltonen et al., 2013), a better estimate of localized tendon strain would still be expected if localized tendon material properties were included in our model. Furthermore, we used a twist of 30°, which has been shown to prevent strain concentrations (Shim et al., 2018), so our estimate of localized strain might be conservative.

Validation of localized strain prediction using strain mapping techniques will be required to confirm the true extent of strain magnitude and heterogeneity in the Achilles tendon. It is however, important to consider that direct measurement of Achilles tendon strain distribution during dynamic task is an extremely challenging task (Maganaris et al., 2017). Franz et al. (2015) provided direct measurements of Achilles tendon displacement during walking; however, tendon displacements were only assessed on a single plane, and within a region of interest of 38 mm within the mid portion of the tendon. Other approaches, such as the use of speckle-tracking based methods under controlled contractile conditions also show potential at this time (Slane and Thelen, 2015). Despite the lack of direct validation of our results, the individual components of our modeling pipeline have been tested and validated (or verified) individually. Our rigid-body EMG-informed NMS model has been used in multiple applications, showing to produce physiologically plausible muscle forces across the lower and upper limbs (Hoang et al., 2018; Kian et al., 2019; Assila et al., 2020; Maniar et al., 2020). Additionally, the personalization via ultrasound and MRI measurements, the calibration of the neuromuscular parameters in the rigid body model, and the tuning of the material properties of the FE model, all contribute to generate physiologically plausible results. Nonetheless, it will be necessary to define some creative ways to further validate and refine our modeling pipeline. Following this methodological evaluation and validation, a detailed survey of interindividual variation of free Achilles tendon strains in a broad range of functional tasks will be of benefit to guide practitioners in prescribing exercise for Achilles tendon training and rehabilitation that are likely to have anabolic or therapeutic benefit. Clinical trials will also be required to demonstrate whether the efficacy of targeted training and rehabilitation informed by real-time feedback of free Achilles tendon strain is enhanced relative to traditional exercise prescriptions where tissue strain is not explicitly targeted.

*In vitro* and *in vivo* studies have identified strain magnitude as an important parameter for maximizing tendon adaptation (Wang, 2006; Arampatzis et al., 2007; Wang et al., 2013, 2015), but more studies are required to better locate the mechanobiological sweet spot for normal as well as pathological tendons. Such studies should explore the effect of different combinations strain magnitude, duration, frequency and rate on tendon remodeling, and identify the optimal combination. A further imperative will be to substantially reduce the time required to generate and run personalized models. A current bottleneck in the process is the time taken to manually segment medical images, which may be addressed in the future using auto-segmentation methods (Cunningham et al., 2016) or statistical shape modeling approaches (Zhang and Besier, 2017). In the context of the present application, statistical shape modeling would involve using machine learning methods to interrogate a large database of 3D tendon geometries to create a personalized 3D tendon geometry based on sparse geometric data obtained from the individual. While no such database currently exists, efforts to do so are recommended.

A further barrier to clinical translation is the current need to directly measure whole body kinematics, ground reaction forces, and muscle activation patterns in order to first calibrate the NMS model and then estimate triceps surae muscle-tendon forces. Although these measurements may be routinely made in a laboratory environment, they are generally not feasible in either the field or a typical clinic. A necessary development will therefore be to develop wearable technologies, such as inertial measurement units to replace marker-based motion capture, which may be imbedded into garments together with wireless EMG sensors. Such systems are already commercially available but would need to be registered to an underlying anatomical model and evaluated against marker-based motion capture systems in terms of their ability to drive a NMS model. Several recent developments also point to the possibility that the current need to directly measure ground reaction forces using a force plate could be eliminated. For example Martin et al. (2018) and Keuler et al. (2019) described a non-invasive method that involves measuring the speed of the of the shear wave generated in response to tapping the tendon to estimate Achilles tendon loading during locomotion. Further, Johnson and colleagues used reduced motion capture marker sets or wearable technology and big data/machine learning to accurately estimate ground reaction forces and moments (Johnson et al., 2018, 2019a,2020) and knee joint moments (Johnson et al., 2019b) from kinematic motion capture data alone. Developments such as these are important because of their potential make direct measurement of ground reaction forces redundant.

## Conclusion

It is clear from both *in vitro* and *in vivo* studies that the Achilles tendon is highly sensitive to its mechanical environment and that there appears to be a strain range that results in positive tendon adaptation. We have demonstrated in this paper that it is possible to generate data that could be used to provide real-time feedback in a training and/or rehabilitation setting to that ensure a targeted dose of mechanical stimuli can be delivered to the Achilles tendon. The main point of difference with this approach, compared to existing approaches in tendon training and rehabilitation, is that the prescribed loading regime for the tendon is defined at the tissue level in terms of localized tendon stresses and/or strains, rather than by whole-body or joint level biomechanics, via a generic set of exercises and dosages. This is an important distinction because (1) the correspondence between external and internal tissue loading is weak due to the high degree of variability between individuals in movement patterns, muscle activation patterns, musculoskeletal geometry and material properties, and (2) defining loading at the level of the tissue (tendon) connects, across size scales, these two previously distinct fields of research, thereby providing the ability to alter exercise prescription to target the mechanobiological sweet-spot for the tendon. We also foresee the potential of targeted training and rehabilitation informed by real-time feedback of localized tissues loading to guide remodeling of other musculoskeletal tissues such as bone, muscle and cartilage, and in the management of other musculoskeletal conditions including osteoarthritis and osteoporosis.

## Data Availability Statement

Upon request, the raw data supporting the conclusions of this article will be made available by the authors, without undue reservation.

## Ethics Statement

The studies involving human participants were reviewed and approved by the Griffith University Human Research Ethics Committee. The participant provided their written informed consent to participate in this study.

## Author Contributions

CP and VS contributed to the model, analyze the data, and conceptualized, drafted, critically revised, and approved the final version of the manuscript. DD contributed to the data collection, and conceptualized, drafted, critically revised, and approved the final version of the manuscript. DL, SO, RN-W, DG, TB, MZ, and RB contributed to the conceptualization, and critically revised and approved the final version of the manuscript. All authors contributed to the article and approved the submitted version.

## Conflict of Interest

The authors declare that the research was conducted in the absence of any commercial or financial relationships that could be construed as a potential conflict of interest.

## Footnotes

1 https://www.cmiss.org
